# Which amount of fluid do we need to evaluate preload dependency?

**DOI:** 10.1186/2197-425X-3-S1-A238

**Published:** 2015-10-01

**Authors:** J Marc, B de Broca, PG Guinot, T Archange, J Garnier, MO Fischer, H Dupont, E Lorne

**Affiliations:** Univ. Hospital Amiens, Amiens, France; CHU Côte de Nacre, Caen, France

## Intr

Cardiac output optimisation is based on stroke volume (SV) variations with fixed volume expansion (VE) or use of dynamic preload indices. The amounts of volume and thresholds used for SV increase vary between studies.

## Objectives

The objective of this study was to evaluate the ability of the stroke volume (SV) variation with 250 ml (Δ250SV) of volume expansion (VE) to predict further SV increase with VE. We also studied the ability of respiratory SV variation (ΔrespSV) to discriminate fluid responisvenness according to the amount of VE (250 then 500 ml). Fluid expansion consisted on infusion of 500 ml of Ringer lactate fractioned by step of 250 ml over 10 min.

## Methods

After IRB approval, 48 patients ventilated with a tidal volume of 7 ml/kg and monitored by an oesophageal Doppler were included. Hemodynamic (heart rate, blood pressure) and ODM (peak velocity (PV), stroke volume (SV), corrected flow time (FTc), cardiac output (CO), ΔrespSV) data were collected before VE, after 250 ml and 500 ml. Responders (R) were defined by an increase ≥10% of SV after VE. We defined three groups : Non-responders (NR), Responders at 250 and 500 ml of VE (R) and Non-responders at 250 ml but Responders at 500 ml of VE (NR250). Data were compared by ANOVA with post-hoc analysis and Mann Whitney test. A ROC curve was constructed for ΔrespSV250, ΔrespSV500 and Δ250SV.

## Results

Of the 48 patients, 18 (38%) were classified as NR, 17 (35%) NR250 and 13 (27%) R. In the overall population, 30 patients increased SV over 10% after 500 ml of VE. In the NR250 group the average Δ250SV was 8% (IC_95_: 6-10) and 11 (23%) patients had a Δ250SV less than 10% whereas they were responders for 500 ml of VE. The Δ250SV AUC was 0,9 (IC_95_:0,79-0,97), p < 0,001, with an optimal threshold at 7%. The ΔrespSV AUC differed with the amount of VE used (0,76(IC_95_:0,62-0,88) for 250 ml and 0,87(IC_95_:0,74-0,95) for 500 ml, p < 0,05).

## Conclusions

In this study, VE with 250 ml of crystalloid solution hid 50% of the responders for 500 ml. For a VE titration, a Δ250SV threshold at 10% could miss an important number of preload dependant patients. Predictability of dynamic preload indices vary according to the amount of VE. It could be related to crystalloid expansion power effect.

